# Assessment of the Kinematic Adaptations in Parkinson’s Disease Using the Gait Profile Score: Influences of Trunk Posture, a Pilot Study

**DOI:** 10.3390/brainsci11121605

**Published:** 2021-12-03

**Authors:** Tauana Callais Franco do Nascimento, Flavia Martins Gervásio, Antonia Pignolo, Guilherme Augusto Santos Bueno, Aline Araújo do Carmo, Darlan Martins Ribeiro, Marco D’Amelio, Felipe Augusto dos Santos Mendes

**Affiliations:** 1 Graduate Program in Rehabilitation Sciences, University of Brasília, Federal District, Brasília 72220-275, DF, Brazil; tauanacallais@gmail.com (T.C.F.d.N.); felipemendes@unb.br (F.A.d.S.M.); 2 Department of Physiotherapy and Physical Education, College of Sport, State University of Goiás, Goiânia 74075-110, GO, Brazil; flavia.gervasio@hotmail.com; 3 Department of Biomedicine, Neurosciences and Advanced Diagnostics, University of Palermo, 90129 Palermo, Italy; marco.damelio@unipa.it; 4 Graduate Program in Sciences and Health Technologies, University of Brasília, Federal District, Brasília 72220-275, DF, Brazil; bueno.guilhermeaugusto@gmail.com; 5 Department of Physiotherapy, University of Brasília, Federal District, Brasília 72220-275, DF, Brazil; aline.adocarmo@gmail.com; 6 Henrique Santillo State Center of Rehabilitation and Readaptation, Goiânia 74653-230, GO, Brazil; darlan.ribeiro@hotmail.com

**Keywords:** posture, gait, Parkinson’s disease, trunk flexion, kinematics

## Abstract

Introduction: Postural abnormalities are common in patients with Parkinson’s disease (PD) and lead to gait abnormalities. Relationships between changes in the trunk posture of PD patients and gait profile score (GPS) and gait spatiotemporal parameters are poorly investigated. The aim of the current study was to investigate the relationships between trunk posture, GPS, and gait spatiotemporal parameters, in patients with PD. Materials and Methods: Twenty-three people with PD and nineteen age-matched healthy people participated in this study. A 3D gait kinematical analysis was applied to all participants using the Plug-In Gait Full Body^TM^ tool. Trunk and limb kinematics patterns and gait spatio-temporal parameters of patients with PD and the control group were compared. Additionally, correlations between trunk kinematics patterns, gait spatio-temporal parameters, and GPS of the PD group were tested. Results: Cadence, opposite foot off, step time, single support, double support, foot off, gait speed, trunk kinematics, and GPS showed significant differences between the two groups (*p* ≤ 0.05). Posture of the trunk during gait was not related to the spatio-temporal parameters and gait profile score in the PD group. The trunk flexor pattern influenced GPS domains, mainly of the ankle and the knee. Discussion and Conclusions: Flexed posture of the trunk in patients with PD seems to influence both ankle and knee movement patterns during the gait. The GPS analysis provided direct and simplified kinematic information for the PD group. These results may have implications for understanding the importance of considering the positioning of the trunk during gait.

## 1. Introduction

Parkinson’s disease (PD) gait is related to difficulty in regulating spatio-temporal and kinematic-angular gait parameters. This is present from the early stages of the disease and progressively contributes to greater difficulty in walking [1,2]. Compared to healthy controls, patients with PD have reduced walking speed [3], step length, and single support, and increased double support time. These modifications are related to the increased risk of falls [4], decreased functionality [5,6], and less independence in performing activities of daily living, and can also be observed in atypical parkinsonisms, such as PSP-P [7]. In people with PD, hip, knee, and ankle joints have a reduced range of motion (RoM). Over time, the ability to move around can be considerably affected, resulting in functional limitations in domains of activity and participation [8]. Additionally, trunk flexion has been recognized as one of the most common postural alterations present in PD [5,9].

From a biomechanical point of view, the trunk represents almost 50% of the body mass [10]. During gait, when flexed, the trunk alters the positioning of the center of mass (CM), reducing the RoM of the hip [11]. The CM displacements are considered as posture destabilizing mechanisms in PD [12]. Few studies evaluate the impact of postural abnormalities, such as trunk flexion, on gait spatio-temporal and kinematic parameters of people with PD [13,14]. Postural deformities found in the intermediate and advanced stages of PD clinical history determine worse gait disorders, postural instability, and functional capacities.

Studies evaluating the influence of trunk positioning and its assessment process, in the early stages of PD, report conflicting results [3,9]. Understanding the impact of trunk positioning on PD gait is of clinical and scientific importance, since changes in trunk positioning are noted during the course of the disease.

In addition to traditional gait measurements, the study of gait quality has been currently being implemented using indexes such as the gait profile score (GPS) [15,16]. The GPS provides a single value summarizing the general deviation of the kinematic data of gait, in relation to normative data, indicating the gait profile by means of a score. This profile is calculated through the analysis of nine kinematic variables, that are assessed bilaterally. The analysis of bilateral kinematic variables results in the gait variable score (GVS) [15], which is expressed in degrees and indicates the joint, the movement, and the lower limb (right and/or left) responsible for the altered GPS [16,17], i.e., GVS highlights each lower limb joint’s, or segment’s, contributions to the GPS. The GPS has been used to assess gait abnormalities in elderly [18], post-stroke [19], cerebral palsy [15], and in minor extension in PD [20] patients.

The characterization of abnormalities in the spatio-temporal parameters and in gait profile, resulting from postural deviation during locomotion, may be relevant to describe different walking patterns and to lead to specific rehabilitation strategies for people with PD. The aims of the present study were to evaluate the presence of postural deviations of the trunk during gait, its relationship with spatio-temporal gait parameters, and the GPS of people with a diagnosis of idiopathic PD. Our hypothesis was that the trunk flexor pattern might influence spatio-temporal gait parameters and the gait profile of patients with PD.

## 2. Materials and Method

### 2.1. Participants

Twenty-three participants with PD currently taking anti-parkinsonian medication and nineteen age-matched controls participated in this study. Inclusion criteria were: (i) clinical diagnosis of PD issued by a neurologist, according to UK Brain Bank criteria, (ii) Hoehn and Yahr stage 1–3 [21], (iii) being in stable treatment with Levodopa, (iv) Mini-Mental State Examination (MMSE) > 24 [22] and Montreal Cognitive Assessment (MoCA) > 26 [23], (v) independent walking without aids, and (vi) to declare not ingesting alcoholic beverages within 24 h prior to the data collection. Exclusion criteria were: (i) presence of previous surgeries in the lower limbs, pelvis, or spine, and (ii) having a medical diagnosis of any other neurological, visual, vestibular, or muscular disorder.

Sample size calculation was performed from a pilot study, considering a 95% confidence interval, a significance level of 0.05, a power of 99%, and considering the null hypothesis (Student’s *t*-test) of the trunk flexion and general GPS measurements between control and PD groups (with effect size, obtained by Cohen’s d test, of 1.93 and 2.23, respectively). The analysis indicated the need for a total of 26 subjects for the trunk flexion variable (13 per group) and 22 subjects for GPS (11 per group). The calculations were performed considering the Student’s *t*-test, using the G* Power software version 3.2 (Universitat Kiel, Germany). Considering a 10% loss, the sample size of 30 subjects was determined for the trunk flexion variable and of 24 subjects for the general GPS variable.

### 2.2. Procedures

A 3D kinematical gait analysis was applied to all participants (people with PD in “on” state of the medication), who walked one 9 meter track at self-selected speed and barefoot. The Plug-In Gait Full Body^TM^ tool, from software Vicon Nexus^®^ version 2.7.1(Vico, Oxford, UK), was used according to the reference guide’s instructions [24]. Data were captured at a frequency of 120 Hz by five Bonita B10 cameras (Vicon Motion Systems Ltd.^®^, Oxford Metrics Group, Oxford, UK) and two other cameras, model Vero v1.3x (Vicon Motion Systems Ltd.^®^, Oxford Metrics Group, Oxford, UK), processed by the fourth-order digital Butterworth filter, applied to the trajectory of the markers, with a cut-off frequency of 10 Hz.

### 2.3. Variables

Five consistent trials from each limb were performed to calculate the average values of spatio-temporal and kinematics variables. Data were exported from Vicon Nexus^®^ software version 2.6.0 (Vicon Motion Systems Ltd.^®^, Oxford Metrics Group, Oxford, UK). The generated data were normalized in function of the gait cycle, using 51 time-normalized samples for each stride. The analyzed spatiotemporal gait parameters were cadence, stride time, opposite foot off, opposite foot contact, step time, single support, double support, foot off, stride length, step length, and walking speed. The GPS and GVS considered the following kinematic variables: pelvic tilt, obliquity, and rotation; hip flexion/extension, abduction/adduction and in-ternal/external rotation; knee flexion/extension; ankle plantar/dorsiflexion; foot pro-gression pelvic tilt, obliquity and rotation; hip flexion/extension, abduction/adduction and internal/external rotation; knee flexion/extension; ankle plantar/dorsiflexion; foot progression.

The GPS represents the analysis of nine kinematic variables (previously mentioned) evaluated bilaterally. The GVS represents the analysis of each of the nine kinematic variables included in GPS [15,16,17], described as right and left side. These scores represent the root mean square differences between patient and reference data. The higher the GPS, the more deviated the gait.

The trunk study considered the clavicle marker as the origin of the coordinate system. The Z-axis is formed between the midpoint of the sternum and T10 to the midpoint of the clavicle and C7. A secondary axis, X, is pointed towards the midpoint of C7 and T10, and the midpoint of the clavicle to sternum. Trunk tilt was measured by the angle formed between the sagittal trunk and the sagittal laboratory axis. A positive value corresponds to trunk flexion. Lateral inclination is measured in the plane of the laboratory transverse axis and the trunk frontal axis, where positive values correspond to inclination to the right. Trunk rotation is calculated over the frontal axis of the thorax coordinate system. It is the angle measured between the sagittal axis of the thorax and the sagittal laboratory axis. Internal rotation is indicated by a positive value [24].

### 2.4. Statistical Analysis

Statistical analysis was performed with SPSS Statistics version 23.0 (IBM, Chicago, IL, USA). The normality of the variables was assessed using the Shapiro–Wilk test, determining the subsequent tests. The sample features were characterized by means of descriptive statistics and reported as mean, standard deviation, and confidence interval. Inferential analysis was performed, comparing the averages of kinematics, spatio-temporal, and GPS, using the Student’s *t*-test for parametric data and the Mann–Whitney test for non-parametric data. The correlation between the angular kinematics of the trunk and the GPS was calculated by Pearson’s correlation. To verify the contribution of the trunk changes on the spatio-temporal parameters, GPS and GVS, simple linear regression was used. The level of statistical significance adopted was *p* ≤ 0.05. A correlation of r ≤ 0.3 was considered “weak”, 0.31 to 0.69 “substantial”, and ≥0.7 “strong” [25].

## 3. Results

Demographic and clinical characteristics of both groups are provided in Table 1. Results show the homogeneity of the groups for the variables age, weight, height, and body mass index.

Significant differences between the two groups were observed for cadence, opposite foot off, step time, single support, double support, foot off, and walking speed (Table 2), as well as for gait profile scores (Table 3).

Analyses of GVS parameters showed significant differences between patients and controls for hip joints (internal and external rotation), knee (flexion and extension), ankle (plantarflexion and dorsiflexion), foot (foot progression angle), and pelvis (anteversion and retroversion) segments, considering both sides (Table 3).

The angular kinematic measures of the trunk indicated a flexor posture pattern for the PD group, and the magnitude of the flexion was significantly different compared to that of controls (Table 4).

The correlation and regression analyses indicated that the trunk flexor pattern could predict differences in GPS domains. There was a strong correlation between trunk flexion and GVS knee variation (left side—r = 0.71, R^2^ = 0.213, *p* = 0.031; right side—r = 0.62, R^2^ = 0.244, *p* = 0, 01) and ankle (left side—r = 0.69, R^2^ = 0.134, *p* = 0.024; right side—r = 0.72, R^2^ = 0.138, *p* = 0.046). The gait speed was not related to the values observed in the GPS and GVS (Table 5).

## 4. Discussion

The current study aimed to evaluate the relationship of the trunk kinematics during gait with the spatio-temporal measurements, gait variable score, and gait profile score of people with PD. The main results pointed to a trunk flexion posture during the gait in the PD group, which stood out as a predictive variable of the kinematic variations of the knee and ankle, providing an altered gait profile.

People with PD present a gait pattern with changes in the spatio-temporal parameters, compromising walking. The main gait feature is slowness, caused by the disease and observed by the reduction of temporal and time-dependent measures, such as gait speed and cadence. These changes are in line with results of other studies showing a reduction in cadence [26,27], step time [28], single support [26,29], and gait speed [5,7,30], and increased double support [31,32], opposite foot-off, and foot-off [26,33,34] in PD. However, the changes found in this study could lead to functional decline [35,36] and an increased risk of falling [7,32].

We found an increase in the GPS (overall, right, and left) in PD, indicating an altered gait profile. This could be explained by the variation found in five of the nine GPS domains (internal/external hip rotation, knee flexion/extension, dorsi/plantar flexion, foot progress, and pelvic obliquity). These angular changes in gait are commonly found in PD and are justified by the reduction in RoM of lower limbs and difficulty in regulating and coordinating movements during gait [26,31,34,37].

The reduced mobility of the ankle in PD reduces the foot initial contact and foot off during gait. In an attempt to avoid flat contact of the foot with the ground and stumbling, hip and knee joints perform compensatory movements during gait [38,39]. Similar changes in GPS and GVS found in our investigation were also observed in Speciali and collaborators´ study carrying out the validation [40] and analysis of GPS in PD during a dual task [41].

The kinematic verification methods used by other studies, investigating angular changes in lower limb joints [37,42,43,44], are not able to describe either gait or quality profile of this population or to identify factors contributing to these changes, such as trunk positioning.

The findings of the kinematic analysis of the trunk highlighted a significant flexor pattern during the gait of participants with a diagnosis of PD, when compared to healthy controls. Such a flexor pattern has been previously reported [5]. Other studies [13,14] also reported the flexor pattern during PD gait, but with higher values, justified by the advanced stages of the disease and deformities already defined, including the Pisa Syndrome and Camptocormia. The trunk plays an important role in the control of posture and movement, and this function can be compromised when the flexor posture is adopted [45].

In PD, the increased trunk flexion and the displacement of the CM, causing changes in the posture, bring about important musculoskeletal adaptations to reduce the risk of falling [5,12]. However, PD is a pathology with increased muscle tone [46,47,48], represented by the tonic hyperactivity of the flexor muscles with co-activation of antagonist muscle groups, rigid joints, and weak and abnormally directed reaction forces on the ground [12]. Thus, a deficient system is recruited to attempt to adjust the posture, providing a trunk flexion and corroborating to increase kinematic variations and the consequent reduction in gait quality.

We identified that the flexed posture during gait could predict the knee and ankle variations, presented by the GVS. These joints, therefore, contribute to an altered gait profile. The reduction in RoM of lower limbs during gait is common in PD, at all stages of the disease [26,42,49,50,51], and it is related to changes in speed [44], increased stiffness [52], and possible compensatory mechanisms [32]. Young people and adults without disease, adopting trunk flexion with different degrees, assume a “crouch” pattern throughout the gait cycle, characterized by sustained knee flexion with increased hip flexion, ankle dorsiflexion at heel strike, and a reduction of plantar flexion in the balance [53,54,55]. The upright posture is mechanically efficient in humans, because the CM displaces over the support surface, like an inverted pendulum [56,57]. Adopting a flexor posture, participants with PD reduce the action of this mechanism. The CM tends to move downward and forward and its oscillations are reduced, as the hip and knee joint amplitude are limited [45]. In healthy individuals, flexed trunk postures may require compensatory changes in kinematics to try to maintain balance during gait.

The findings highlighted that changes in the trunk angulation, although smaller than those observed in other studies, contributed to the increase in the angular variation of the knee and ankle. Gait speed is considered a key variable for gait analysis [58], even though no contribution has been observed on the GPS and GVS. Our findings support the hypothesis that GPS could gain information about the quality of gait even if there is no modification in key spatio-temporal variables. This hypothesis was partially proven in the current study, when there was an absence of a relationship between temporal variables and the GPS.

Contribution of the postural pattern, to the authors’ knowledge, was not addressed in the analysis of the GPS/GVS. The trunk analysis must be considered during the evaluation of scientific studies and in clinical practice for a better interpretation of the results and creating a therapeutic approach. The study of the gait profile requires an inspection of the global and individual joint kinematics, in addition to other typical characteristics of people with PD, such as the flexor pattern during walking.

Our study has some limitations. First, it is a cross-sectional study, where the data must be analyzed apart from the causal relationship. A second limitation is that the analysis was restricted to the trunk and lower limbs. In the future, more relationships from other segments, such as the head and upper limbs, should be incorporated into the study of motor symptoms in people with PD.

As strengths, this study highlights the importance of understanding the mechanisms responsible for adopting stooped posture in people with PD and the relationship with gait parameters, which may be relevant to guide better gait rehabilitation procedures in this population.

## 5. Conclusions

In this pilot study, our findings showed that the kinematic analysis of the trunk indicated a flexion pattern during gait in the PD group, and this posture contributed to worse GVS for the knee and ankle. Therefore, trunk posture could lead to angular adaptations of the knee and ankle joints. These adaptations could contribute to a greater GPS, reflecting worse gait quality in people with PD.

## 6. Study Limitations

A limitation of this study may be the relatively small sample size in each group, which may affect the statistical power of the analyses.

## Figures and Tables

**Table 1:** Characteristics of the PDG (*n* = 23) and CG groups (*n* = 19).

		PDG	CG	
		Mean (SD)	Mean (SD)	*p*
Age (years)		63.74 (±5.98)	64.37 (±4.32)	0.703
Weight (kg)		67.85 (±6.34)	66.26 (±6.38)	0.572 ^a^
Height (meters)		1.66 (±0.05)	1.64 (±0.06)	0.334
BMI (kg/m^2^)		24.51 (±1.17)	25.52 (±1.75)	0.626
MoCA (score)		27.82 (±1.46)	29.42 (±0.76)	<0.001 *
Mini-Mental (score)		28.17 (±1.72)	29.63 (±0.68)	0.001 *
Diagnostic time (years)		4.64 (±4.01)		
Hoehn and Yahr stage	Stage 1	11 (47.84%)	11 (47.84%)	
	Stage 2	9 (39.13%)	9 (39.13%)	
	Stage 3	3 (13.04%)	3 (13.04%)	

Note: ^a^—Comparative analysis performed by Student’s *t*-test and Mann–Whitney test; *—significance of *p* ≤ 0.05; PDG—Parkinson’s disease group; CG—control group; kg—kilogram; kg/m^2^—kilogram/meter square; MoCA—Montreal Cognitive Assessment.

**Table 2:** Comparison of the spatio-temporal parameters between PDG (*n* = 23) and CG (*n* = 19) groups.

	PDG	CG	
	Mean (SD)	Mean (SD)	*p*
Cadence (steps/min)	104.94 (±9.02)	111.43 (±7.16)	0.015 *
Stride time (s)	1.14 (±0.09)	1.10 (±0.10)	0.153
Opposite foot off (%)	14.51(±3.32)	10.17 (±2.27)	<0.001 *
Opposite foot contact (%)	50.09 (±0.52)	50.14 (±0.70)	0.791
Step time (s)	0.57 (±0.05)	0.54 (±0.03)	0.010 *
Single support (s)	0.41 (±0.04)	0.43 (±0.03)	0.031 *
Double support (s)	0.33 (±0.09)	0.24 (±0.05)	<0.001 *
Foot off (%)	64.16 (±3.45)	61.10 (±1.52)	0.001 *
Stride length (m)	1.08 (±0.13)	1.10 (±0.13)	0.552
Step length (m)	0.54 (±0.06)	0.56 (±0.06)	0.333
Walking speed (m/s)	0.94 (±0.15)	1.04 (±0.14)	0.024 *

Note: Comparative analysis performed by Student’s *t*-test. *—Significance of *p* ≤ 0.05; PDG—Parkinson’s disease group; CG—control group; steps/min—steps per minute; s—seconds; m—meters; m/s—meters per second; SD—standard deviation.

**Table 3:** Comparison of the GPS and GVS parameters between PDG (*n* = 23) and CG (*n* = 19) groups.

	PDG	CG	
	Mean (SD)	Mean (SD)	*p*
GPS (degree)			
Overall	8.22 (±1.54)	5.11 (±1.16)	<0.001 *
Left	7.72 (±2.04)	4.74 (±1.03)	<0.001 *
Right	7.66 (±1.23)	4.86 (±1.59)	<0.001 *
GVS			
Pelvic tilt	5.43 (±3.93)	4.15 (±2.81)	0.241
Hip flex/ext left	7.92 (±5.32)	6.00 (±3.24)	0.176
Knee flex/ext left	8.13 (±2.81)	6.65 (±2.57)	0.044 *
Ankle dorsi/plan left	5.61 (±2.98)	3.56 (±1.58)	0.010 *
Pelvic obliquity	2.37 (±1.77)	1.32 (±0.68)	0.019 *
Hip add/abd left	5.05 (±4.19)	3.47 (±2.20)	0.147
Pelvic rotation	3.13 (±1.43)	2.51 (±1.46)	0.170
Hip rotation int/ext left	13.74 (±2.88)	3.03 (±4.51)	<0.001 *
Foot progression left	6.52 (±4.88)	4.25 (±1.14)	0.044 *
Hip flex/ext right	7.88 (±4.40)	6.02 (±3.69)	0.151
Knee flex/ext right	7.89 (±2.77)	6.48 (±3.14)	0.030 *
Ankle dorsi/plan right	5.85 (±3.21)	3.73 (±1.98)	0.016 *
Hip add/abd right	4.89 (±4.64)	3.40 (±2.03)	0.202
Hip rotation int/ext right	13.84 (±2.06)	4.33 (±5.28)	<0.001 *
Foot progression right	5.92 (±2.46)	4.46 (±1.01)	0.020 *

Note: Comparative analysis performed by Student’s *t*-test; *—Significance of *p* ≤ 0.05; PDG—Parkinson’s disease group; CG—control group; GPS—gait profile score; GVS—gait variable score; Hip flex/ext—hip flexion/extension; Knee flex/ext—knee flexion/extension; Ankle dorsi/plan—ankle dorsi/plantarflexion; Hip abd/add—hip adduction/abduction; SD—standard deviation; Pelvic tilt = pelvic inclination in the sagital plane; Pelvic obliquity = pelvic inclination in the frontal plane; Pelvic rotation = pelvic rotation considering the transverse plane; Hip rotation int/ext right: right hip joint angle in the transverse plane; Hip rotation int/ext left: left hip joint angle in the transverse plane; Foot progression right = rotation of the right lower extremity in the transverse plane; Foot progression left= rotation of the left lower extremity in the transverse plane.

**Table 4:** Comparison of the kinematic trunk parameters between PDG (*n* = 23) and CG (*n* = 19) groups.

	PDG	CG	
	Mean (SD)	Mean (SD)	*p*
**Trunk kinematics (degree)**			
Flexion/extension mean	4.03 (±2.62)	0.72 (±0.72)	<0.001 *
Flexion/extension maximum	5.33 (±3.18)	1.31 (±1.36)	<0.001 *
Flexion/extension minimum	3.43 (±3.28)	−0.94 (±1.53)	<0.001 *
Lateral inclination mean	0.01 (±0.07)	−0.01 (±0.11)	0.390
Lateral inclination maximum	1.44 (±0.62)	1.18 (±0.52)	0.166
Lateral inclination minimum	−1.38 (±0.65)	−1.20 (±0.62)	0.380
Rotation mean	−0.05 (±0.20)	−0.06 (±0.19)	0.843
Rotation maximum	2.13 (±0.96)	2.54 (±0.86)	0.158
Rotation minimum	−2.30 (±0.82)	−2.64 (±0.76)	0.171

Note: Comparative analysis performed by Student’s *t*-test; *—Significance of *p* ≤ 0.05; PDG—Parkinson’s disease group; CG—control group; SD—standard deviation.

**Table 5:** Correlation of the trunk kinematics and the gait speed with GPS parameters and GVS of the PDG (*n* = 23).

	Trunk Flexion Mean			Trunk Flexion Maximum			Trunk Flexion Minimum			Walking Speed		
	r	R²	*p*	r	R²	*p*	r	R²	*p*	r	R²	*p*
GPS (degree)												
Overall	0.24	0.13	0.27	0.26	0.024	0.227	0.19	−0.007	0.371	0.084	−0.04	0.704
Left	0.21	0.002	0.311	0.23	0.008	0.288	0.17	−0.017	0.433	0.08	−0.041	0.716
Right	0.13	−0.029	0.539	0.21	−0.001	0.335	0.16	−0.021	0.465	0.043	−0.046	0.847
GVS												
Pelvic tilt	0.01	−0.047	0.944	0.14	−0.025	0.504	0.11	−0.033	0.591	−0.134	−0.029	0.542
Hip flex/ext left	−0.01	−0.047	0.948	0.12	−0.031	0.571	0.07	−0.042	0.729	−0.246	0.016	0.259
Hip flex/ext right	0.38	0.108	0.069	0.31	0.057	0.142	0.25	0.023	0.232	−0.07	−0.042	0.750
Knee flex/ext left	0.71	0.213	0.031 *	0.86	0.245	0.026 *	−0.86	−0.045	0.826	−0.052	−0.045	0.813
Knee flex/ext right	0.62	0.244	0.010 *	0.77	0.219	0.022 *	−0.85	0.168	0.330	−0.114	−0.034	0.604
Ankle dorsi/plan left	0.69	0.134	0.024 *	0.85	0.19	0.001 *	−0.74	−0.045	0.835	0.037	−0.046	0.867
Ankle dorsi/plan right	0.72	0.138	0.046 *	0.85	0.192	0.021 *	0.15	0.056	0.144	−0.337	0.071	0.116
Pelvic obliquity	−0.10	−0.038	0.648	−0.11	−0.033	0.595	−0.09	−0.039	0.679	0.191	−0.01	0.384
Hip add/abd left	−0.06	−0.043	0.771	−0.06	−0.043	0.764	−0.07	−0.042	0.732	0.552	0.271	0.066
Hip add/abd right	0.04	−0.045	0.831	0.09	−0.038	0.670	0.11	−0.034	0.609	0.379	0.103	0.075
Pelvic rotation	−0.46	0.176	0.241	−0.5	0.276	0.141	−0.47	0.192	0.210	0.221	0.003	0.312
Hip rotation int/ext left	0.38	0.108	0.069	0.31	0.057	0.142	0.25	0.023	0.232	−0.07	−0.042	0.750
Hip rotation int/ext right	0	−0.048	0.990	−0.02	−0.047	0.917	−0.04	−0.046	0.846	0.095	−0.038	0.667
Foot progression left	0.24	0.017	0.254	0.15	−0.021	0.471	0.19	−0.010	0.385	0.10	−0.037	0.650
Foot progression right	−0.07	−0.042	0.745	0.13	−0.03	0.552	0.19	−0.009	0.378	−0.081	−0.041	0.714

Note: Pearson correlation analysis, linear regression; *—Significance of *p* ≤ 0.05; PDG—Parkinson’s disease group; GPS—gait profile score; GVS—gait variable score; Hip flex/ext—hip flexion/extension; Knee flex/ext—knee flexion/extension; Ankle dorsi/plan—ankle dorsi/plantarflexion; Hip abd/add—hip adduction/abduction; Pelvic tilt = pelvic inclination in the sagital plane; Pelvic obliquity = pelvic inclination in the frontal plane; Pelvic rotation = pelvic rotation considering the transverse plane; Hip rotation int/ext right: right hip joint angle in the transverse plane; Hip rotation int/ext left: left hip joint angle in the transverse plane; Foot progression right = rotation of the right lower extremity in the transverse plane; Foot progression left = rotation of the left lower extremity in the transverse plane.

## Data Availability

All data generated or analyzed during this study are included in this article. Further enquiries about information on the data generated and analyzed can be directed to Felipe Augusto Dos Santos Mendes.
